# Assessment of prevalence of preeclampsia from Dilla region of Ethiopia

**DOI:** 10.1186/s13104-015-1821-5

**Published:** 2015-12-24

**Authors:** Prabhanjan Kumar Vata, Nitin M. Chauhan, Arasumani Nallathambi, Fentaw Hussein

**Affiliations:** College of Health Sciences, Dilla University, 419 Dilla, SNNPR Ethiopia; College of Natural and Computational Sciences, Dilla University, 419 Dilla, SNNPR Ethiopia

**Keywords:** Preeclampsia, Hypertension, Proteinurea, Quality care, Neonatal morbidity, Maternal morbidity, Ethiopia

## Abstract

**Background:**

Preeclampsia is a multi-organ system disorder that occurs after the 20th week of gestation in pregnancy and is characterized by hypertension and proteinuria. In Africa more than 270,000 women die from maternal deaths, worldwide approximately 76,000 women and 500,000 babies die yearly due to preeclampsia. It affects about 8–10 % of all pregnancies. Studies have shown that up to 77 % women affected with preeclampsia lack knowledge about preeclampsia, and therefore cannot take preventative measures. The aim of study is to evaluate the outcomes and quality of care given to preeclamptic patients treated in Dilla University Referral Hospital.

**Methods:**

The study is a retrospective, hospital based study. One hundred and seventy two records of women were retained for final study out of 7702 patients from January 2009 to December 2012.

**Results:**

The incidence rate of preeclampsia in Dilla University Referral Hospital was found to be 2.23 %. The common mean ages found to be affected for preeclampsia were 19.2, 22.5 and 27.8 and 31.5 with a trend towards increasing severity with younger age population.

**Conclusion:**

A guideline on the management and prevention of preeclampsia needs to be produced for Ehiopia.

## Background

Preeclampsia has been a major cause of poor result in pregnancy and the category “hypertensive diseases of pregnancy” and is a leading cause of maternity in Africa [1]. It is a common pregnancy disorder that correspond one out of the three cases i.e. obstetric morbidity, hypertension and/or proteinuria [2, 3]. Preeclampsia is the most public hazardous pregnancy complication affecting mother as well as foetus [4]. It is assorted with an increased risk factor of cardiovascular diseases and type II diabetes in later life cycle of mother [5]. Preeclampsia is responsible for approximately 50,000 maternal deaths worldwide annually out of which 25 % of cases are due to Intra Uterine Growth Restrictions (IUGR) and 15 % are outcome of preterm birth in developed countries [4]. However, there is no known cure for preeclampsia apart from delivery of the baby and the placenta. Accordingly, early diagnosis of preeclampsia and close observation are assertive for controlling preeclampsia during pregnancy.

The strategy employed by high-income countries have successfully reduced both the incidence of eclampsia and the mortality associated with it by almost 90 %, utilizing a combination of early detection during Antenata Care and increased access to hospital care for women who develop preeclampsia [6]. Parallel reduction mostly in rural China and Srilanka suggest that this framework for routinely screening pregnant women for hypertension and proteinuria, treating severe preeclampsia patients with anti-hypertensive and anti-convulsant drugs and, if necessary, ending the pregnancy by inducing childbed or dealing caesarean delivery can work as good model especially in low-income countries [7]. In Africa, preeclampsia occurs in 10 % of pregnancies, which is significantly higher than the global average of approximately 2 % [1]. Roughly, four in every hundred women develops problem of high blood pressure and leaky kidney during pregnancy. Black women are more prone to get high blood pressure and preeclampsia than white women [8]. The saving mother report on confidential enquiry in maternal death from South Africa (1999–2001) reported poor quality of care being a major contributor to maternal death from preeclampsia. The deaths were attributed to lack of transport (11–20 %); lack of appropriately trained medical staff (up to 55 %) and failure in recognize patient’s problems (12 %). In addition to this, 64 % of the preeclampsia women who died in 1998 and 55.5 % of those who died in 2001 had received sub-standard management [9]. There are very few studies that report the prevalence of preeclampsia in Africa [1, 10, 11]. According to a 2010 UN report, Ethiopia is one of the five countries that together account for 50 percent of the world’s maternal deaths and rural areas mostly contribute to maternal deaths [12]. In 2011, the country recorded 676 maternal deaths for every 100,000 live births, up from 673 in 2005. Unfortunately, the reason for such death is still unclear. There is no in-depth study so far done in Ethiopia, mostly in Dilla region, which looks at the extent of “confirmed” preeclampsia and its contribution to the maternal and perinatal morbidity and mortality.

The objective of this study is to evaluate the outcome and quality of care given to preeclampsia patients treated in Dilla University Referral Hospital of Ethiopia within the period of January 2009 to December 2012 by using observational study.

## Methodology

### Study area and design

Retrospective study was conducted from January 2009 to December 2012 to assess the prevalence of eclampsia among patients diagnosed at Dilla University Referral Hospital. Dilla town is the capital of Gideon Zone situated in SNNPR state with a total area of 1347.4 square kilometres and encompasses two cities administrations with six woredas. It is located at an altitude of 1300–3000 m above sea level and the climate favours desert conditions. The total population is 94,189 out of which 46,058 (49.9 %) are males and 48,131 (50.1 %) are females [13].

The Dilla University Referral Hospital is the only tertiary care institute that received referral of high and low risk pregnancies from village hospitals, general practitioners and specialist obstetricians in addition to their own patients. Different senior medical superintendents, 19 physicians, 53 nurses lead the referral hospital with a bed capacity of 156.

### Ethical consideration and source of data

It was believed that the study will contribute to the provision of quality care to preeclamaptic women, permission to conduct the study was granted by the Higher Degree Committee of the University Referral Hospital. After obtaining the consent from higher authorities the data was collected from register book. As the study consisted entirely of confidential record review, no informed consent was deemed necessary. Precautions were taken to keep personal identifier data separate from data collected using confidential study numbers. Information on demographic, clinical, laboratory and management of all preeclamptic patients and their babies who were admitted to referral hospital during the study period was retrieved from the maternity admission registers, delivery books and operation books.

### Sampling method and size

From January 2009 to December 2012 approximately there were 7702 patients admitted in gynaecology ward of Dilla University Referral Hospital. Women with a diagnosis or listed complications of preeclampsia or eclampsia, or symptoms associated with preeclampsia (i.e. hypertension, convulsions, oedema, etc.) were selected from the maternity and theatre register books during the study period. The latter (symptoms) were included to improve complete case finding. These files were systematically retrieved and reviewed for the following inclusion criteria {standard definition for diagnosis of preeclampsia: blood pressure >140/90, proteinuria > +1, with or without oedema} [14]. Due to potential loss or unavailability of older records, cases were selected in backward fashion from three years of hospital maternity and theatre registers and records until the desired sample size was obtained. Those with symptoms but not confirmed diagnosis and others who did not registered according to criteria were excluded.

### Quality care for patients

The quality of care was measured based on the basis of technical care received by study subjects during their stay in the hospital. The quality of care that has been given to the patients at different levels (during ante partum, stages of labour, and when complication were observed) including appropriateness of medications and mode of delivery was measured against the standard management protocols for preeclampsia as recommended by the South African National Department of Health and National Committee for the Confidential Enquiry into Maternal Deaths [15].

A total of 172 cases confirmed of preeclampsia were included during the study period to demonstrate neonatal and maternal outcomes. The salient features included name and age of patients, obstetric history, present illness, the findings of general physical examination and systemic examination specially abdominal and vaginal examination. Patients were assessed on the basis of history, clinical examination, ultrasound and laboratory investigations during the study interval.

### Statistical interference of data

The data was processed using Statistical Software Packages for Social Science (SPSS) by using Chi square test. *P* < 0.05 was considered statistically significant.

## Results

### Incidence of preeclampsia

**Fig. 1:**
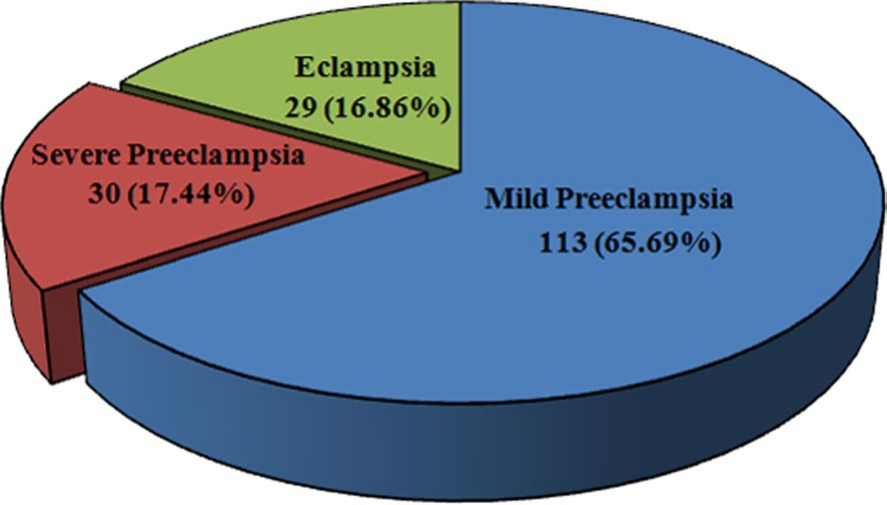
Distribution and frequency of different categories of preeclmaptic cases of Dilla University Referral Hospital from January 2009 to December 2012

Generally, 7702 patients were admitted to gynecology ward out of which 172 admissions were preeclamptic. On the basis of this data the incidence rate of preeclampsia in Dilla University Referral Hospital was found to be 2.23 %. Out of 172 preeclmaptic women with the diagnosis of preeclampsia, 113 (65.68 %) had mild preeclampsia, 30 (17.42 %) posses severe preeclampsia and remaining i.e. 29 (16.83 %) showed eclampsia (Fig. 1).

### Socio-demographic data

**Fig. 2:**
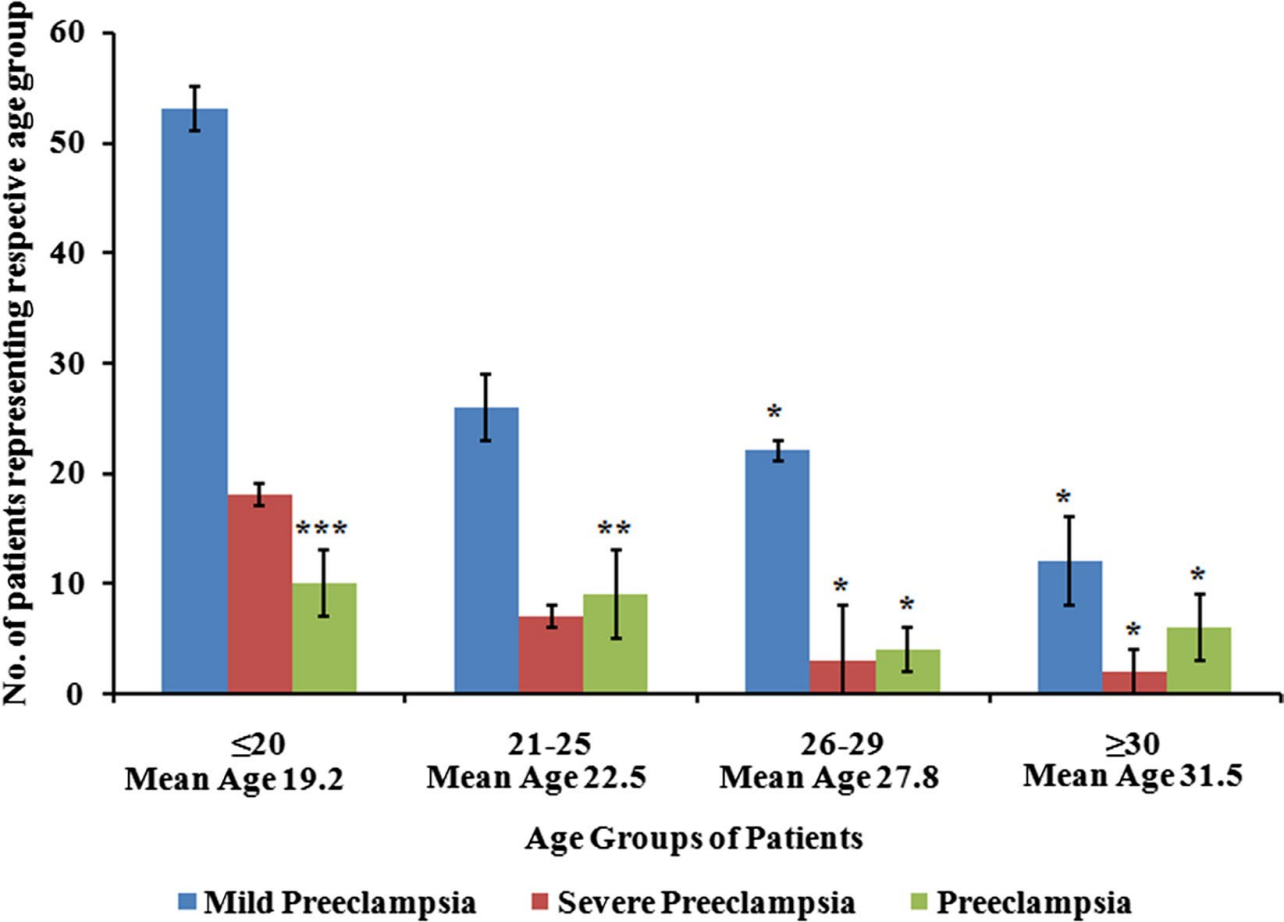
Age group of preclamptic cases registered in Dilla University Referral Hospital from January 2009 to December 2012. Values indicated are the distribution of preeclamptic patients to different age groups noted in Dilla University Referral Hospital. *Error bar* represents standard deviation. ***Highly significant; **moderate significant; *less significant

The common mean ages found to be affected for preeclampsia were 19.2, 22.5 and 27.8 and 31.5. Among 172 suffered patients, 81 (47.09 %) were in mean age group of 19.2. While, 42 (24.41 %) belongs to 22.5 mean age group. 29 patients (16.86 %) out of 172 suffered women represent to 27.8 mean group of age and the rest i.e. 20 (11.63 %) has 31.5 mean age group (Fig. 2). These mean differences were significant (*P* = 0.0005, *P* = 0.0023, *P* = 0.0032, *P* = 0.0046 for 19.2, 22.5, 27.8, 31.8 mean age group respectively) and suggested a trend towards increasing severity with younger age population. Ethnically out of 172 patients 61 (35.46 %), 42 (24.41 %), 29 (16.86 %), 21 (12.20 %), 19 (11.04 %) were belongs to Amhara, Sidama, Welayata, Oromo and Gurage respectively (Table 1).

**Table 1:** Distribution of different categories of preeclampsia among different ethnic category of Dilla region

Ethnic Group	Number of patients			Total
	Mild preeclampsia	Severe preeclampsia	Eclampsia	
Amhara	44 (25.58 %)	11 (06.39 %)	06 (03.48 %)	61 (35.46 %)
Sidama	32 (18.60 %)	06 (03.48 %)	04 (02.32 %)	42 (24.41 %)
Welayata	17 (09.88 %)	10 (05.81 %)	02 (01.16 %)	29 (16.86 %)
Oromo	09 (05.23 %)	02 (01.16 %)	10 (05.81 %)	21 (12.20 %)
Gurage	11 (06.39 %)	01 (00.58 %)	07 (04.06 %)	19 (11.04 %)
Total	113 (65.68 %)	30 (17.42 %)	29 (16.83 %)	172

Values indicated are the distribution of preeclamptic patients to different ethnic group. Values in parenthesis indicates the frequency of percentage of three different categories of preeclampsia

### Quality control of patients from Dilla University Referral Hospital

On the basis of Dilla University Referral Hospital, length of stay of suffered patients were 1–5 days for 79 (45.93 %), 6–10 days for 54 (31.39 %), 11–15 days for 23 (13.37 %), 16–20 days for 11 (6.39 %) and 21–25 days for 5 (2.90 %) of all the preeclamptic women. The mean hospital stay was 6.0 days for the mild preeclamptic, 8.3 days for the severe preeclamptic and 9.1 days for that of eclamptic. On admission to hospital 80 (46.51 %) out of all the patients had no physical complaints, while 51 (29.65 %) had a headache, 12 (6.97 %) epigastric pain, 15 (8.72 %) convulsion, 8 (4.65 %) blurred vision and 6 (3.48 %) vaginal bleeding (Table 2).

**Table 2:** Quality control background characteristics of 172 preeclamptic patients admitted to Dilla University Referral Hospital

	Number of patients			Total
	Mild preeclampsia	Severe preeclampsia	Eclampsia	
Stay in Hospital				
1–5 days	67 (38.95 %)	08 (04.65 %)	04 (02.32 %)	79 (45.93 %)
6–10 days	36 (20.93 %)	13 (07.55 %)	05 (02.90 %)	54 (31.39 %)
11–15 days	07 (04.06 %)	04 (02.32 %)	12 (06.97 %)	23 (13.37 %)
16–20 days	02 (01.16 %)	04 (02.32 %)	05 (02.90 %)	11 (06.39 %)
21–25 days	01 (00.58 %)	01 (00.58 %)	03 (01.74 %)	05 (02.90 %)
Total	113 (65.68 %)	30 (17.42 %)	29 (16.83 %)	172
Complaints				
No physical complaints	71 (41.27 %)	06 (03.48 %)	03 (01.74 %)	80 (46.51 %)
Headache	34 (19.76 %)	15 (08.72 %)	02 (01.16 %)	51 (29.65 %)
Epigestric pain	02 (01.16 %)	06 (03.48 %)	04 (02.32 %)	12 (06.97 %)
Convulsion	02 (01.16 %)	01 (00.58 %)	12 (06.97 %)	15 (08.72 %)
Blurred vision	03 (01.74 %)	01 (00.58 %)	04 (02.32 %)	08 (04.65 %)
Vaginal bleeding	01 (00.58 %)	01 (00.58 %)	04 (02.32 %)	06 (03.48 %)
Total	113 (65.68 %)	30 (17.42 %)	29 (16.83 %)	172
Gestation week				
22–25	03 (01.74 %)	02 (01.16 %)	01 (00.58 %)	06 (03.48 %)
26–30	17 (09.88 %)	01 (00.58 %)	02 (01.16 %)	20 (11.62 %)
31–35	36 (20.93 %)	13 (07.55 %)	14 (08.13 %)	63 (36.62 %)
36–40	55 (31.97 %)	13 (07.55 %)	12 (06.97 %)	80 (46.51 %)
41 and above	02 (01.16 %)	01 (00.58 %)	00 (00.00 %)	03 (01.74 %)
Total	113 (65.68 %)	30 (17.42 %)	29 (16.83 %)	172
Mode of delivery				
SVD	14 (08.18 %)	04 (02.32 %)	01 (00.58 %)	19 (11.04 %)
C-Section	71 (41.27 %)	61 (35.46 %)	21 (12.20 %)	153 (88.95 %)
Total	85 (49.41 %)	65 (37.79 %)	22 (12.79 %)	172 (100 %)
Indication of C-section				
Mild preeclampsia	22 (14.37 %)	24 (15.68 %)	07 (04.57 %)	53 (34.64 %)
Severe preeclmapsia	22 (14.37 %)	12 (07.84 %)	08 (05.22 %)	42 (27.45 %)
Foetal distress	13 (08.49 %)	12 (07.84 %)	02 (01.30 %)	27 (17.64 %)
Eclampsia	05 (03.26 %)	07 (04.57 %)	01 (00.65 %)	13 (08.49 %)
Previous C-section	05 (03.26 %)	03 (01.74 %)	02 (01.30 %)	10 (06.53 %)
Failed induction	02 (01.30 %)	02 (01.30 %)	01 (00.65 %)	05 (03.26 %)
Abruptio placenta	01 (00.65 %)	01 (00.58 %)	00 (00.00 %)	02 (01.30 %)
IUFD	01 (00.65 %)	00	00 (00.00 %)	01 (00.65 %)
Total	71 (46.40 %)	61 (39.86 %)	21 (13.72 %)	153 (100 %)
Neonatal variables				
Live birth	13 (7.55 %)	119 (69.18 %)	24 (13.95 %)	156 (90.69 %)
Still Birth	02 (1.16 %)	11 (6.39 %)	3 (1.74 %)	16 (9.30 %)
Low birth weight	5 (2.90 %)	12 (6.97 %)	6 (3.48 %)	23 (13.37 %)

Values indicated are the distribution of preeclamptic patients to different quality control given to patients admitted to Dilla University Referral Hospital. Values in parenthesis indicates the frequency of percentage of three different categories of preeclampsia

Gestational age on admission to referral hospital was found to be 6 (3.48 %) at 22–25 weeks, 20 (11.62 %) at 26–30 weeks, 63 (36.62 %) at 31-35 weeks, 80 (46.51 %) at 36–40 weeks and 3 (1.744 %) at 41+ weeks. The mean gestational age for the mild preeclamptic was 34.8 weeks, 33.1 weeks for severe preeclamptic and for eclamptic it was 35.3 (Table 2).

One hundred fifty three (88.93 %) of the preeclamptic women of the study group gave birth by means of caesarean section and nineteen (11.07 %) vaginally (Table 2). The indication for caesarean deliveries were: 53 (34.64 %) mild preeclampsia, 42 (27.45 %) severe preeclampsia, 27 (17.64 %) foetal distress, 13 (8.49 %) eclampsia, 10 (6.43 %) previous caesarean section, 5 (3.26 %) failed induction, 2 (1.30 %) abruptio placenta, and 1(0.65 %) intra uterine foetal death (IUFD) (Table 2).

The results regarding the perinatal conditions showed a prevalence of 90.69 % of live births. Low birth weight (<2.5 kg) was seen in 13.37 % of cases. There were 9.30 % of stillbirths (Table 2). Perinatal mortality rate increased as severity of preeclampsia increased. Neonatal intensive care unit admissions were seen in 19.4 % of cases.

## Discussion

Anticipation of preeclampsia is widely depends on detection of risk factors, measurement of arterial pressure, proteinuria and edema. The importance of anticipating which women will develop preeclampsia lies in the need of particular medical care and preventive measures that might prolong the pregnancy and reduced the maternal and foetal risks [16]. Majority of preeclampsia related deaths in the low income countries widely occurs in community, thus it must be focussed at this level. Especially in Africa, preeclampsia occurs in 10 % of pregnancies, which is significant higher than the global average of approximately 2 % [1]. It is important that preventive strategies must be applied to every pregnant woman since it is behind the scope to predict which women will develop preeclampsia. Three primary delays can lead to increased in relative incidence of maternal mortality from preeclampsia includes: late in decision making to attempt, delay in approaching health facility and detain in health service provision. The prevalence of preeclampsia observed in the proposed study is in the agreement with the data found in African countries, where approximately 10 % of total pregnancies cases are diagnose with preeclampsia. The frequency of preeclampsia which is around 2.23 % almost equivalent to the expected value of 2 % quoted in global literature [1].

Our study demonstrates that there was no significant maternal mortality observed during the study period. The neonatal mortality was also low in comparison to maternal mortality (Table 2). It was also found that approximately 925 of all the preeclamptic patients received anti-hypertension drugs. Some of interesting findings are encouraging when it was noticed that: there was no significant difference in the management of different categories of preeclampsia studied; caesarean section was extremely high (88.93 %); use of magnesium sulphate as prophylaxis was very low (only 15 % of severe preeclampsia cases received magnesium sulphate) (result not shown).

Out of 173 women, 152 (87.86 %) preeclamptic women in the study group were in the range of age group of 16-30 years indicating younger women are more prone to preeclampsia in comparison to elder one. Results of ages are correlates to the studies of Moodley and Mashioane [17] and Brown and Buddle [18]. Significant differences in the mean age of different categories of preeclampsia suggest a trend towards increasing severity with younger age which also corresponds to the findings of Hall et al. [19] where younger women are at higher risk for developing eclampsia. The decrease incidence of preeclampsia by ethnicity from Amhara to Gurage could be due to the distribution of population among Dilla region. Similarly when ethnicity is compared by colour Amhara, Sidama amd Welayata which are black in colour contribute more to preeclampsia (76.73 %) compare to other ethnic group (Table 1). Thus our studies indicates that being black is a risk factor for preeclampsia. Apart from above results there was no significant difference (*P* = 0.613) in the mean hospital stay between three categories of preeclampsia of our study. This indicates that there was little distinction in the management of difference types of preeclampsia.

Different studies have shown that prolongation of gestation in uncomplicated preeclampsia enhances foetal maturity [20]. Similarity of the mean hospital stay and almost universal delivery by caesarean among different patients in this study indicates that there was not proper management practice in Dilla University Referral Hospital. Most of the mild preeclampsia reports can be managed expectantly as frequent recommended management of mild preeclampsia [21] and in return may reduced Intensive Care Unit (ICU) admission of neonates. However, other studies also shown that aggressive management comparison with expectant management results in equivalent maternal morbidity, fewer small for gestation age infants and marks the seriousness for neonatal morbidity [22].

In the end the findings indicates that the management of preeclampsia in Dilla University Referral Hospital lacks specificity for different categories of preeclampsia. This could be because of lacks of guidelines in the management of preeclampsia to assist clinicians to make critical decision when they are challenge by different severity of diseases. Further research on risk factors may suggest effective ways to prevent preeclampsia.

## Conclusions

The results presented are the systematic information for care given to preeclamptic cases was not totally in line with the international or South African regional guidelines of preeclampsia management. A guideline on the management and prevention of preeclampsia needs to be produced for Ehiopia, and further research on risk factors and on cost effectiveness of premature termination of pregnancy needs to be look seriously.

## References

[1] Nakimuli A, Chazara O, Byamugisha J, Elliott AM, Kaleebu P, Mirembe F, Moffett A (2013). Pregnancy, parturition and preeclampsia in women of African ancestry. Am J Obstet Gynecol.

[2] Hirose A, Borchert M, Niksear H, Alkozai AS, Gardiner J, Fillippi V (2012). The role of care-seeking delays in intrauterine fetal deaths among ‘near-miss’ women in Herat, Afghanistan. Paediatr Perinat Epidemiol.

[3] Duley L (1992). Maternal mortality associated with hypertensive disorders of pregnancy in Africa, Asia, Latin America and the Caribbean. Br J Obstet Gynaecol.

[4] Bell MJ (2010). A historical overview of preeclampsia-eclampsia. J Obstet Gynecol Neonatal Nurs.

[5] Lindheimer MD, Umans JG (2006). Explaining and predicting preeclampsia. N Engl J Med.

[6] Kim YM, Ansari N, Kols A, Tappis H, Currie S, Zainullah P, Bailey P, Roosmalen J, Stekelenburg J (2013). Prevention and management of severe preeclampsia/eclampsia in Afganistan. BMC Pregnancy Child Birth.

[7] Ronsmans C, Campbell O (2011). Quantifying the fall in mortality associated with interventions related to hypertensive diseases of pregnancy. BMC Public Health.

[8] Jacobs DJ, Vreeburg SA, Dekker GA, Heard AR, Priest KR, Chan A (2003). Risk factors for hypertension during pregnancy in South Australia. Aust N Z J Obstet Gynaecol.

[9] Pattinson B, Saving M. Second report on confidential enquiries into maternal deaths in South Africa (1999–2001). 2002, 117–8.

[10] Osungbade KO, Olusimbo KI (2011). Public health perspectives of preeclampsia in developing countries: implication for health system strengthening. J Pregnancy.

[11] Murphy DJ, Stirrat GM (2000). Mortality and morbidity associated with early onset pre-eclampsia. Hypertens Pregnancy.

[12] Gaym A, Bailey P, Pearson L, Admasu K, Gebrehiwot Y (2011). Disease burden due to pre-eclampsia/eclampsia and the Ethiopian health system’s response. Int J Obstet Gynaecol.

[13] Dennis PH, Berhanu. From social identity and community in the fertility of southern Ethiopian women. In: Samuel A, John BC, editors. Reproduction and social context in sub-Saharan Africa. A Collection of Micro-Demographic Studies. Westport, Connecticut: Greenwood Press; 2003.

[14] Beaulieu MD: Prevention of pre-eclampsia. Canadian Guide to clinical preventive Health care. Ottawa: Health Canada 1994, pp. 136–143.

[15] Guidelines for Maternity Care in South Africa. A manual for clinics, Community Health Centres and District Hospitals. 2002, pp. 70–83.

[16] Brown MC, Bell R, Collins C, Waring G, Robson SC, Waugh J, Finch T (2013). Women’s perception of future risk following pregnancies complicated by preeclampsia. Hypertens Pregnancy.

[17] Mashiloane CD, Moodley J (2002). Induction or caesarean section for pre-term preeclampsia. J Obstet Gynaecol.

[18] Brown MA, Buddle M (1996). Hypertension in pregnancy: maternal and neonatal outcome according to laboratory and clinical features. Med J Aust.

[19] Hall DR, Odendaal HJ, Kristen GF, Smith J, Grove D (2000). Expectant management of early onset severe pre-eclampsia: perinatal outcome. BJOG.

[20] Lydakis C, Beevers M, Beevers DG, Lip GY (2001). The prevalence of pre-eclampsia and obstetric outcome in pregnancies of normotensive and hypertensive women attending a hospital specialist clinic. Int J Clin Pract.

[21] Magee LA, Dadelzen P, Bohun CM, Rey E, El-Zibdeh M, Stalker S (2003). Serious perinatal complications of non-proteinuric hypertension: an international multicentre, retrospective cohorts study. J Obstet Gynaecol Can.

[22] Allen VM, Joseph KS, Murphy KE, Magee LA, Ohlsson A (2004). The effect of hypertensive disorders in pregnancy on small for gestational age and stillbirth: a population based study. BMC Pregnancy Childbirth.

